# Transcultural adaptation and validation of the Eating Self-Efficacy Brief Scale (ESEBS): the Brazilian version

**DOI:** 10.1007/s40519-024-01703-2

**Published:** 2024-11-04

**Authors:** Ana Maria Pandolfo Feoli, Tainá Lopes da Silva, Janete de Souza Urbanetto, Monica D’Amico, Silvia Cerolini, Caterina Lombardo

**Affiliations:** 1 Eating Behavior Group of the Psychology Postgraduate Program, School of Health and Life Sciences, Pontifical Catholic University of Rio Grande do sul, Porto Alegre, 90619-900 Brazil; 2 School of Health and Life Sciences, Pontifical Catholic University of Rio Grande do sul, Ipiranga Avenue 6681 – Partenon, Porto Alegre, RS 90619-900 Brazil; 3 Department of Psychology, Sapienza University of Rome, Via Dei Marsi 78, 2nd Floor, 00185 Rome, Italy; 4 Department of Human Sciences, Guglielmo Marconi University, Via Plinio 44, 00196 Rome, Italy

**Keywords:** Eating self-efficacy, Eating behavior, Scale, Validation

## Abstract

**Purpose:**

This study aimed to develop and validate the Brief Eating Self-Efficacy Scale (ESEBS-BR) in Brazilian Portuguese, addressing the lack of tools to assess eating self-efficacy beliefs in Portuguese-speaking populations.

**Method:**

The study sought to cross-culturally adapt the scale, evaluate its internal structure, validate its reliability and validity, and explore potential associations between eating self-efficacy and eating behaviors. The cross-cultural adaptation process involved translation and back-translation, expert committees, and pre-testing. Scale validation was conducted with 228 participants, including reliability analyses, confirmatory factor analysis, and correlations with established measures of eating behavior.

**Results:**

The ESEBS-BR, maintaining all 8 original items, demonstrated high reliability, with a two-factor structure model confirmed by confirmatory factor analysis. Significant correlations were found between ESEBS-BR scores and measures of binge eating and eating behaviors, validating its criterion validity.

**Conclusions:**

The development and validation of the ESEBS-BR represent a significant contribution to the assessment of eating self-efficacy in the Brazilian population. The scale proved to be sensitive, reliable, and valid, offering an important tool for research and clinical interventions related to eating behavior.

*Level of evidence*: V, descriptive cross-sectional study.

**Supplementary Information:**

The online version contains supplementary material available at 10.1007/s40519-024-01703-2.

## Introduction

Eating self-efficacy has been referred to as the belief in one’s ability to self-regulate eating behavior [1–3]. The concept of self-efficacy was initially introduced by Bandura within his social-cognitive theory of human behavior (e.g. 1997) and refers to personal beliefs related to specific behavioral domains [4]. Self-efficacy was found to have a role in promoting behavioral change in primary care [5] and health behaviors [6].

Having a short but valid and reliable instrument for assessing eating self-efficacy is relevant for many reasons. First of all, it has often been reported that personal belief in one’s ability to adopt a certain behavior is a good predictor of the frequency of adoption of that behavior [7]. Self-regulating eating behavior is essential for both promoting healthy eating and counteracting overeating. Studies have shown that adherence to the Mediterranean diet, one of the most acknowledged healthy eating patterns so far, is predicted by self-efficacy [8, 9]. Self-efficacy also predicted higher scores on the Healthy Eating Index (e.g. [10]) and acted as a mediator in explaining healthy eating (i.e., eating more fruits and vegetables) after 2 weeks [11]. Oikarinen and colleagues [12] found that low eating self-efficacy is linked to unfavorable eating behaviors, including cognitive restraint, uncontrolled eating, emotional eating, and binge eating. Moreover, several studies reported reduced eating self-efficacy in individuals with eating disorders, such as Binge-Eating Disorder (BED), when facing situations characterized by negative emotions or physical discomfort, as compared to people without this diagnosis (e.g. [13–15]).

Most scales used for evaluating eating self-efficacy refer to the beliefs in one's ability to regulate dieting or include questions related to dieting, weight management, overeating, and restriction of caloric consumption and are primarily used in contexts evaluating weight loss interventions (e.g. [16, 17]). In 2020, a scale to assess self-efficacy in regulating eating in emotional and social contexts was developed and validated using a sample from the Sapienza University of Rome community in Italy (the Eating Self-Efficacy Scale, ESEBS [18]. This scale has the advantage of assessing personal belief in regulating eating behavior without referring to dieting or weight loss and using a reduced number of items (only 8); four measured emotional self-efficacy, and the other four measured social self-efficacy. The sample used for validation included 137 men and 273 women, with a mean age of 31.73 years (SD = 10.78) and a mean BMI of 22.73 (SD = 3.81). Showing good psychometric properties and internal consistency. So far, no similar instruments in the Brazilian Portuguese language have been validated. Adapting a questionnaire to a new context is considered the best approach to ensure equivalent metrics across different populations. This method preserves conceptual and methodological integrity, allows meaningful comparisons, and ensures consistency in data collection in multinational studies while minimizing selection bias due to the lack of translated versions.

In light of the potential usefulness in different healthcare contexts, due to its brevity, content, and properties, we decided to adapt the scale to Portuguese. The aim of the present study is to report on the psychometric properties of the Brazilian Portuguese version of the Eating Self Efficacy Brief Scale (ESEBS-BR).

## Method

The sample was one of convenience, recruited through social media, including participants of both sexes aged 18 and over, with the exclusion of those who did not correctly complete any stage of the study between June and July 2023. Sample size calculation was conducted according to Pasquali’s recommendations [19]. It was considered to include 15 respondents per each of the 8 items constituting ESEBS-BR, increasing by 20% for possible missing data. This resulted in a minimum size of 144 participants in order to get reliable results from the parametric tests. Data were collected from a total of 241 participants. However, 13 individuals who completed the questionnaires incorrectly or incompletely were excluded from the study, bringing the total to 228 participants finally included in the study and analysis.

### Ethical procedures

The study was conducted in accordance with the Declaration of Helsinki and approved by the Research Ethics Committee of the Pontifical Catholic University (CAAE: 64659222.0.0000.5336). Participants were invited to voluntarily take part in the study without any form of compensation. They completed the forms and questionnaires only after confirming their understanding and agreeing to the Informed Consent Form.

### Stage 1—transcultural adaptation

The transcultural adaptation process followed the model proposed by Beaton et al. [20], consisting of six stages. It began with translation, where two translators fluent in Italian and with Portuguese as their native language, one specialized in the field and the other without knowledge of the theme, independently translated the original version into Portuguese, generating versions T1 and T2. The T1 and T2 versions were compared and synthesized by an impartial researcher, mediating discussions on translation differences resulting in a common translation (T-12). The T-12 version was back-translated into Italian by two translators, unaware of the tool's purpose, ensuring correspondence with the original version and generating versions RT1 and RT2.

Two committees were formed; the first unified all versions into a pre-final version in Portuguese, and the second, composed of specialists from different regions of Brazil, analyzed equivalences in four levels: semantic, idiomatic, experiential/cultural, and conceptual. The scale was pre-tested on a sample of 30 participants, following Beaton et al. [20] recommendations. Participants responded to sociodemographic questionnaires and the pre-final version of the ESEBS-BR, providing feedback on clarity and understanding of the items.

All produced documents were reviewed by the responsible researchers, and the adapted version was sent to the original authors for approval. The analysis of content validity was based on experts' responses regarding semantic, idiomatic, experiential, and conceptual equivalences. The content validity index (CVI) was calculated based on a minimum agreement of 0.80 among judges, verifying the scale's validity [21, 22].

### Stage 2—ESEBS-BR validation and psychometric analyses

#### Questionnaires

Participants completed the questionnaires online on the QualtricsXM platform (www.qualtrics.com). The survey included the transcultural version of ESEBS adapted for Brazilian Portuguese (ESEBS-BR), other validated measures of eating behavior, and sociodemographic and anthropometric information. Completing the survey took about 15 min. The following instruments were used for this stage:

- Sociodemographic and anthropometric data: A brief survey was specifically created for this research and contained questions on age, gender, weight, height, education level, marital status, living situation, and monthly family income. Body Mass Index (BMI) was calculated based on self-reported data as follows: weight in kilograms (kg) divided by height in meters (m) squared (kg/m^2^).
- ESEBS-BR, 8 items, Brazilian version: This tool is a self-report scale consisting of 8 items that aim to measure how easy it would be to resist the urge to eat in two different situations on a 6-point Likert response scale ranging from 0 “not easy at all” to 5 “completely easy” [18]. The original version of the scale consists of two subscales, namely social and emotional: the former measures the ability to regulate eating in social contexts (e.g., "*How easy would it be for you to resist the urge to eat when eating out with friends*"), and the latter, the ability to resist in situations of emotional activation (e.g., "*How easy would it be for you to resist the urge to eat when you are worried about work/study reasons*”). Cronbach’s alphas from the original study revealed good reliabilities, namely being 0.820 for the Emotional scale and 0.786 for the Social scale.
- Binge Eating Scale (BES): In this tool, participants are required to choose the statement that most accurately reflects their response for each item. Each statement is associated with a score ranging from 0 to 3, covering the spectrum from absence ('0') to maximum severity ('3') of the BED. The cumulative score is determined by summing the points assigned to each item [23, 24]. Following the instructions given by Marcus et al. [25], individuals are categorized based on the following scores: those with a score equal to or less than 17 are classified as not having BED; those with scores between 18 and 26 are classified as having moderate BED; and those with a score equal to or greater than 27 are identified as having severe BED. In the present study, Cronbach's alpha was 0.89.
- The Three Factor Eating Questionnaire (TFEQ-21): To determine the degrees of cognitive restriction, emotional eating, and lack of eating control, the classification instructions provided by the team that developed the questionnaire [26] were used. A 4-point response format was used for items 1 to 20, and an 8-point numerical rating scale was used for question 21 [26]. The Cronbach's statistics in our sample were for the Emotional scale 0.93, Cognitive Restriction 0.75, and Lack of Control 0.84.

### Data analyses

Data were analyzed with JASP versions 0.14.1.0 and 0.19 and Jamovi 2.3.26.

To assess the normality of the distribution, skewness and kurtosis values were considered, accepting a distribution with skewness and kurtosis lower than the absolute value of 1 as normal, that is a value between −1 and + 1 [27].

A Confirmatory Factor Analysis (CFA) was performed to test the internal structure of the ESEBS-BR. In accordance with the original study [18], a model with two correlated factors was tested. The model fit was evaluated using the Maximum Likelihood (ML) Chi-square test statistic, Comparative Fit Index (CFI), Tucker Lewis Index (TLI), Standardized Root Mean Square Residual (SRMR), and Root Mean Square Error of Approximation (RMSEA). By convention, a CFI and TLI > 0.900 indicate an adequate model fit, and a CFI and TLI > 0.950 indicate a good fit. An SRMR < 0.08 is considered a good fit. RMSEA values < 0.050 represent a close fit, while values between 0.050 and 0.080 represent a reasonably close fit, and values > 0.080 represent an unacceptable fit [28]. In order to examine the invariance of the measurement across gender, multigroup CFAs were performed. First, configural invariance was tested, maintaining the same pattern of free and fixed loadings. Secondly, metric invariance was explored by constraining factor loadings to be equal across males and females. Finally, scalar invariance was tested by fixing the equality of factor loadings and intercepts across genders. To ascertain measurement invariance, changes in CFI, RMSEA, and SRMR, were calculated. Consistently with Cheung and Rensvold [29] and Chen [30], ΔCFI ≤ 0.01, ΔRMSEA ≤ 0.015, ΔSRMR ≤ 0.010 were considered acceptable changes. Reliability was measured using Cronbach's alpha and McDonald's omega tests with 95% confidence intervals. Values are considered adequate when ≥ 0.70 [31]. Pearson’s correlation test assessed the criterion validity between ESEBS-BR, TFEQ, and BES. BES was chosen consistently with the original study due to the reported co-occurrence between BED symptoms and lack of eating self-efficacy [13–15]. The TEFQ scales, especially the subscales assessing uncontrolled eating and emotional eating, share common features with the two factors of ESEBS-BR. Furthermore, these features often overlap in individuals with eating disorders and reduced eating self-efficacy [12]. Average Variance Extracted (AVE) and Composite Reliability (CR) were calculated to assess convergent validity. Values were considered acceptable according to Fornell and Larcker’s criteria (AVE ≥ 0.5; CR ≥ 0.70) [32].

Six subgroups were formed based on participants’ BMI using the World Health Organization’s (WHO) norms: underweight participants, BMI < 18.5; normal weight participants, BMI between 18.5 and 24.9 kg/m^2^; participants with overweight, BMI between 25.0 and 29.9 kg/m^2^; participants with first-class obesity, BMI between 30.0 and 34.9 kg/m^2^; second class obesity, BMI between 35.0 and 39.9 kg/m^2^; and third class obesity BMI > 40.0 kg/m^2^. Emotional and social ESEBS scale scores were used to compare groups through a one-way ANalysis Of Variance (ANOVA). Due to missing data, some participants were excluded from some of the analyses as follows: Correlations were calculated on 190 participants who completed all the questionnaires included in the analyses; ANOVAs were performed on 185 participants. Individuals with underweight were excluded due to the very small subsample size (n = 4). For the same reason, I, II, and III class obesity were combined into a single “Obesity” group. In order to ensure the appropriateness of the analyses, normality was assessed in each group for both ESEBS-BR in emotional and ESEBS-BR in social contexts; moreover, variance in BMI groups was assessed through Levene’s test. The results were interpreted with a 95% confidence interval. Tuckey’s Honestly Significant Difference (HSD) correction was applied to Post Hoc tests to control the probability of Type I errors [33].

## Results

### Stage 1—transcultural adaptation

To assess content validity, the Content Validity Index (CVI) was utilized. This index measures the proportion or percentage of judges’ agreement on specific aspects of the questionnaire or its items. Judges were provided with the questions of the ESEBS-BR and had to indicate the fitness of each item regarding semantic, idiomatic, cultural, and conceptual appropriateness on a scale from one to four, going from “*not equivalent*” to “*absolutely equivalent*.” The CVI was then calculated for each item by summing experts’ responses rated "3" and "4" and dividing the sum by the total number of responses. The maximum expected score for the CVI is 1, corresponding to a rate of 4 from each judge. In our analysis, all eight items achieved the maximum content validity score, indicating that all evaluated items have satisfactory equivalence in the analyzed dimensions and content validity, according to the experts' responses.

### Stage 2—validation and psychometric analyses

#### Sample characteristics

The sample included in the final version of the study consisted of 228 individuals, 179 of which were women (78.51%), with a mean age of 38.54 years (Standard Deviation, SD = 13.45) and a mean BMI of 26.65 kg/m^2^ (SD = 5.62). Table 1 presents the characteristics of the sample. The final sample was mainly composed of participants from the Southern region (i.e., 90%). Few participants completed the questionnaires from the Southeastern (6.25%), Northern (2.50%), and Central-Western (1.25%) regions. There were no participants from the Northeastern region.

**Table 1:** Characteristics of the validation study sample

	N
Total sample	228
Mean age (SD)	38.54 (13.45)
Body Mass Index (kg/m^2^) (SD)	26.65 (5.62)
BMI classification N (%)	
Underweight (< 18.5 kg/m^2^)	6 (2.63%)
Normal (18,5–24,9 kg/m^2^)	102 (44.73%)
Overweight (25,0–29,9 kg/m^2^)	68 (29.82%)
Obesity class I (30,0–34,9 kg/m^2^)	40 (17.54%)
Obesity class II (35.0–39.9 kg/m^2^)	9 (3.94%)
Obesity class III (≥ 40 kg/m^2^)	3 (1.31%)
Civil status N (%)	
Single	96 (42.11%)
Married	79 (34.65%)
Widower	4 (1.75%)
Living with partner	35 (15.35%)
No information	14 (6.14%)
Family income N (%)	
No income	4 (1.77%)
Up to 1 minimum wage	5 (2.21%)
1 to 3 minimum wages	30 (13.27%)
3 to 6 minimum wages	52 (23.01%)
6 to 9 minimum wages	49 (21.68%)
9 to 12 minimum wages	34 (15.04%)
12 to 15 minimum wages	21 (9.29%)
More than 15 minimum wages	31 (13.72%)
No information	2
Highest education N (%)	
Elementary unfinished	1 (0.44%)
Elementary finished	0
High school unfinished	1 (0.44%)
High school finished	14 (6.14%)
University unfinished	43 (18.86%)
University finished	55 (24.12%)
Postgraduate	114 (50.0%)

The skewness and kurtosis analyses indicated that the data were normally distributed according to the Empiric Criteria of ± 1.

### Internal structure: Confirmatory Factor Analysis (CFA)

**Fig. 1:**
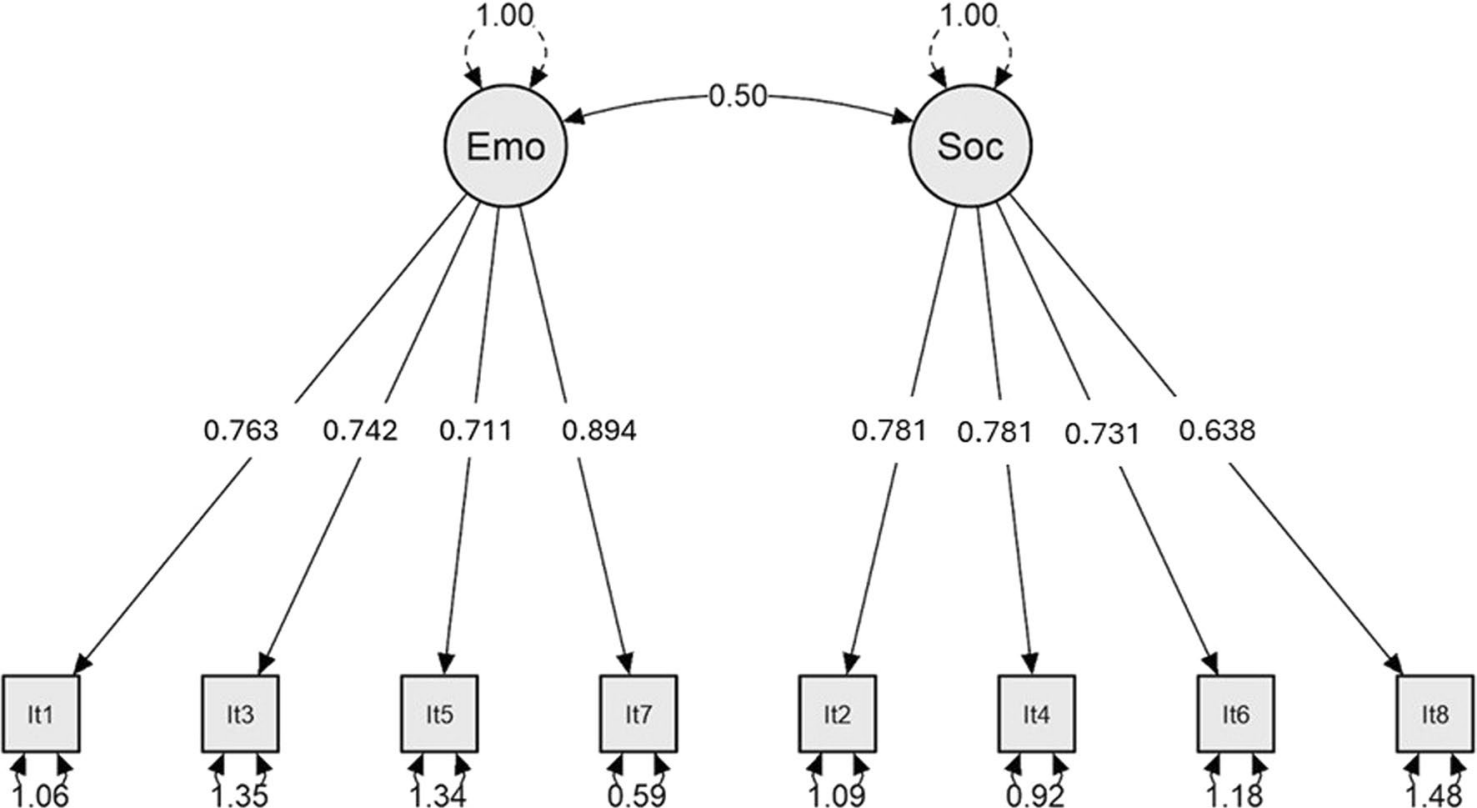
CFA: Factorial loadings of the two-factor model for the items in the ESEBS-BR

The results of the CFA for ESEBS-BR confirm a good fit with the two-factor model (χ^2^(19) = 35.354; p = 0.013; RMSEA ≤ 0.068 [95% CI 0.031–0.103]; SRMR = 0.036; TLI = 0.962; CFI = 0.974). The standardized loadings ranged from 0.638 to 0.894. The factors’ covariance was 0.50 (p < 0.001). Figure 1 shows the standardized loadings of CFA for the items in the ESEBS-BR. The original two-factor model confirmed a good fit in the Brazilian version of the instrument as supported by the observed data.

### Multigroup analysis

The invariance of the ESEBS across genders assessed by multigroup CFAs showed a good fit of the configural model (see Table 2). Metric invariance was confirmed (ΔCFI = 0.001, ΔRMSEA = 0.003, ΔSRMR ≤ 0.008), as well as scalar invariance (ΔCFI = 0.001, ΔRMSEA = 0.003, ΔSRMR ≤ 0.008).

**Table 2:** Fit index of configural, metric, scalar, and strict invariance over gender

Invariance hypothesis	CFI	ΔCFI	RMSEA	ΔRMSEA	SRMR	ΔSRMR
Configural	0.964		0.080		0.048	
Metric	0.963	0.001	0.077	0.003	0.056	−0.008
Scalar	0.965	−0.002	0.070	0.007	0.057	−0.001

*CFI* Comparative Fit Index, *RMSEA* Root Mean Square Error of Approximation, *SRMR* Standardized Root Mean Square Residual

### Reliability

The internal consistency for the Social scale was high (Cronbach's α = 0.822, 95% CI 0.776–0.861; McDonald's ω = 0.824, 95% CI 0.783–0.866), as was for the Emotional scale (Cronbach's α = 0.858, 95% CI 0.821–0.889; McDonald's ω = 0.861, 95% CI 0.829–0.894).

### Criterion and convergent validity

For the correlation analyses, 190 participants yielded valid scores and thus were included. The raw scores for the Emotional and Social subscales of ESEBS-BR were calculated by summing the scores of the four items for each scale in accordance with the results of the factorial analyses (see Table 3). Pearson’s correlation test of eating self-efficacy in emotional context and social contexts with the Binge Eating Scale (BES) and the Three-factor Eating Questionnaire (TFEQ) identified a moderate negative correlation between scores on the ESEBS-BR in the emotional scale and BES scores (r = −0.526, p < 0.001). In contrast, high scores on the ESEBS-BR in the social scale corresponded to low scores on the BES with a weak intensity (r = −0.281, p < 0.001).

**Table 3:** Correlation between self-efficacy, BES, TFEQ dimensions, and BMI

	BES	TFEQ emotional eating	TFEQ cognitive restraint	TFEQ disinhibition	BMI
Emotional ESEBS-BR	−0.526*	−0.687*	−0.182	−0.464*	−0.275*
Social ESEBS-BR	−0.281*	−0.285*	−0.079	−0.335*	−0.102

*BES* Binge Eating Scale, *TFEQ* Three-factor Eating Questionnaire, *BMI* Body Mass Index, *ESEBS-BR* Eating Self Efficacy Brief Scale – Brazilian Version

^*^p ≤ 0.001

Regarding the dimensions of the TFEQ, scores on the ESEBS-BR in internal contexts exhibited a moderate to strong negative correlation with the emotional eating dimension of the TFEQ (−0.687, p < 0.001). Concerning the uncontrolled eating scale (disinhibition), the ESEBS-BR score in both emotional and social scales showed a moderate negative correlation (−0.464 and −0.335, respectively, p < 0.001) with the TFEQ score in Table 3. Partial correlations were still significant when controlling for BMI (all *p*_s_ < 0.001). Both ESEBS-BR scales demonstrated good AVE and CR (Emotional: AVE = 0.610, CR = 0.861; Social: AVE = 0.540, CR = 0.824).

The ANOVAs were performed on 185 participants due to missing data. ESEBS-BR in emotional contexts and ESEBS-BR in social contexts were normally distributed according to the Empiric Criteria of ± 1 in each subgroup (see Table 4). Homoscedastic assumptions were respected for both analyses. Levene’s test was not significant for ESEBS-BR in emotional contexts (F = 0.442; p = 0.643) nor for ESEBS-BR in social contexts (F = 0.253; p = 0.777).

**Table 4:** Skewness and Kurtosis of emotional and social ESEBS-BR

BMI subgroups	N	Emotional ESEBS-BR		Social ESEBS-BR	
		Skewness	Kurtosis	Skewness	Kurtosis
Normal weight	83	−0.165	−0.760	0.028	−0.610
Overweight	58	0.242	−0.791	−0.147	−0.637
Obesity	44	0.312	−0.971	0.559	−0.471

*ESEBS-BR* Eating Self-Efficacy Brief Scale Brazilian Version, *BMI* Body Mass Index, *N* Number of subjects

Results showed significant differences between the three groups for eating self-efficacy in emotional contexts (F_(2,182)_ = 5.598; p = 0.004; ω = 0.047), but not in social contexts (F_(2,182)_ = 1.664; p = 0.192; ω = 0.007). More specifically, post-hoc showed that there was a significant difference in emotional ESEBS-BR between obesity and overweight (t = −2.442; p_tuckey_ = 0.041; d = −0.639) and between obesity and normal weight (t = −3.302; p_tuckey_ = 0.003; d = −0.487) with a medium effect size. People with obesity reported significantly less eating self-efficacy than people with overweight and people with normal weight (see Table 4). No significant difference in ESEBS-BR was found between overweight and normal weight (t = 0.745; p_tuckey_ = 0.737; d = 0.124). Post-hoc for ESEBS-BR in social contexts confirmed that there were no significant differences between obesity and overweight (t =−1.824; p_tuckey_ = 0.165; d = −0.361), between obesity and normal weight (t = −1.153; p_tuckey_ = 0.483; d = −0.213) nor between overweight and normal weight (t = −0.874; p_tuckey_ = 0.657; d = -0.152). The mean scores for each subgroup are described in Table 5.

**Table 5:** Descriptive analyses of emotional and social ESEBS-BR mean across the BMI subgroups

Subgroups BMI	Emotional ESEBS-BR			Social ESEBS-BR		
	Mean	SD	N	Mean	SD	N
Normal weight	10.940	5.073	83	9.265	5.073	83
Overweight	10.241	5.054	58	10.034	5.054	58
Obesity	7.568	5.391	44	8.159	5.391	44

*ESEBS-BR* Eating Self-Efficacy Brief Scale Brazilian Version, *BMI* Body Mass Index, *SD* Standard Deviation

## Discussion

This study successfully developed, adapted, and psychometrically validated the Brazilian Portuguese version of the ESEBS, which we named the “Escala Breve de Autoeficácia Alimentar (ESEBS-BR)”. This adapted tool retains all 8 original items in the same order. The translated version demonstrated semantic, idiomatic, cultural, and conceptual appropriateness for the Brazilian population and Portuguese language while also exhibiting high reliability and strong psychometric properties.

The original version of the ESEBS [18] differs from earlier tools designed to evaluate eating self-efficacy through several distinctive features and no similar instrument is available in the Brazilian Portuguese language so far. Its principal strength is to direct attention towards the examination of eating self-efficacy beliefs in diverse scenarios, as opposed to the main focus of most of the already available scales, such as dieting, adopting a healthy eating regimen, or managing weight loss.

After translating and adapting the original instrument in Portuguese for the Brazilian population, the second stage of the study utilized Confirmatory Factor Analysis (CFA) to empirically validate the hypothesized two-factor model for the scale, consistent with the original. The results not only affirmed the internal consistency of the ESEBS-BR but also underscored its reliability. Multigroup CFAs confirmed the invariance of ESEBS-BR across genders for configural, metric and scalar models, showing that males and females appeared to conceptualize the construct and the items in the same way. However, these results should be interpreted cautiously due to the difference in the group size when comparing genders.

Latent correlations with previously established measures like BES and TFEQ supported the criterion validity of the ESEBS-BR. Specifically, the two sets of scale scores of the ESEBS-BR exhibited a negative correlation with indicators of binge eating and disinhibition, emotional eating, and cognitive restraint. The well-established connection between eating self-efficacy and eating behaviors, as well as psychological traits associated with eating disorders, has been extensively documented in prior research [3, 34, 35]. Additionally, previous studies reported an association between the utilization of dysfunctional strategies to regulate emotions and an increase in food intake [35–37]. The moderate correlation observed between the ESEBS-BR emotional domain and emotional eating domain from TFEQ suggests that while perceived efficacy in regulating eating behavior in emotional situations may predict emotional eating, it likely represents a distinct construct from what was initially conceptualized when the scale was developed. This finding underscores the complexity of eating behavior regulation and highlights the need for further exploration into the complex relationship between these constructs.

Moreover, the absence of significant correlations between both scales of the ESEBS-BR and the Cognitive Restraint domain of TFEQ is encouraging. This suggests that perceived efficacy in regulating eating behavior differs from perceived control over eating behavior. Such differentiation enhances our understanding of the multifaceted nature of eating behavior and reinforces the validity of the ESEBS-BR as a tool for assessing specific aspects of eating self-regulation.

Furthermore, consistent negative correlations were observed between the emotional subscale of the ESEBS-BR and binge eating symptoms, as measured by the BES. The study conducted by Chao et al. [15] found that among patients seeking bariatric surgery, those diagnosed with Binge Eating Disorder (BED) had significantly lower scores on the eating self-efficacy subscales, including control over negative emotions and social pressure, compared to those without BED.

Based on the results obtained, the study examined the differences in mean scores of two variables, emotional and social eating self-efficacy, among three subgroups categorized by BMI: normal weight, overweight, and obesity. The subdivision based on BMI criteria allowed for a more nuanced understanding of how weight status might influence these variables. Interestingly, when comparing the mean scores of the emotional subscale of the ESEBS-BR among the subgroups, a statistically significant difference was observed, indicating that individuals with different BMI statuses exhibit variations in emotional eating self-efficacy may vary with BMI. Specifically, in our sample, people with obesity exhibited lower eating self-efficacy in emotional contexts than people with overweight and people with normal weight, while there were no significant differences between people with normal weight and people with overweight. This finding suggests a potential relationship between weight status and emotional eating self-efficacy, warranting further investigation into the underlying mechanisms comprising the presence of ED diagnosis that may influence such a relationship. Conversely, when assessing the mean scores of the social subscale among the BMI groups, no statistically significant difference was found, implying that weight status may not have a significant impact on social eating self-efficacy or that this difference is so small that it does not emerge with our relatively small sample. However, it is essential to interpret these results with caution and consider potential confounding variables that may influence the relationship between BMI and psychological variables.

The study has limitations, particularly the sample's composition, which was predominantly female (78.51%) and highly educated (50% with postgraduate degrees), limiting the generalizability of the findings. This educational profile is notably higher than the national average, where only 18.6% of the Brazilian population holds a college degree (IBGE, 2023). Future research should validate the Eating Self-Efficacy Brief Scale (ESEBS-BR) in more diverse populations to enhance its broader applicability consistently with the cultural diversity of Brazil. Additionally, all data was collected through self-administered surveys, which may introduce biases such as social desirability. To improve the generalizability of our findings, it is recommended that future research endeavors recruit participants from a wider range of socioeconomic backgrounds and from different settings beyond social media platforms, including community centers and healthcare institutions. Adopting this more inclusive approach will provide deeper insights into the scale’s applicability and relevance across diverse segments of the Brazilian population, thereby enhancing the robustness of the research outcomes. Second, the cross-sectional design of this study restricts our ability to infer causality between eating self-efficacy and eating behaviors. This study was conducted at a single point in time. A longitudinal design is recommended to assess the stability of the ESEBS-BR over time. Tracking participants over several months would allow us to monitor changes in self-efficacy related to eating behaviors, confirm test re-test reliability, and determine the scale’s sensitivity to behavioral interventions aimed at improving eating habits. Finally, the use of self-reported weight and height to calculate BMI may lead to variations in values; however, with the rise of online studies, self-reported weight and height are being used more frequently. Previous research indicated that these measurements are as reliable as those obtained through direct measurement [38–40].

The ESEBS-BR fills an important gap by providing a reliable and psychometric valid brief tool to assess eating self-efficacy beliefs in the Brazilian population. Analysis of subgroups categorized by Body Mass Index (BMI) revealed significant differences in ESE levels, while correlations between scale dimensions and established measures supported its validity.

The relationships found in the present study not only validate the criterion validity of the ESEBS-BR but also suggest potential clinical applications. For instance, the scale could be used to identify individuals at higher risk for binge eating behaviors, enabling early intervention. Further research could explore the utility of ESEBS-BR in therapeutic settings, examining whether improvements in self-efficacy scores can predict better outcomes in eating disorder treatments.

These findings contribute to a better understanding of eating self-efficacy and its implications for interventions aimed at promoting healthy eating behaviors. However, further studies are needed to explore the applicability and utility of the ESEBS-BR in different contexts and populations, as well as to investigate its relationship with long-term dietary health outcomes.

### What is already known on this subject?

- The ESEBS-BR maintains all 8 original items in order and demonstrates high reliability and psychometric validity.
- The ESEBS focuses on examining eating self-efficacy beliefs across various scenarios rather than solely focusing on dieting, adopting a healthy eating regimen, or managing weight.
- The connections found with other well-known measures like BES and TFEQ confirmed that the ESEBS-BR accurately assesses eating self-efficacy.
- The brevity of ESEBS-BR enables its utilization in both clinical and research environments.

### What does this study add?

- The present study aimed to validate the Brazilian Portuguese version of the ESEBS scale.
- The correlation analysis between the ESEBS-BR and established measures such as BES and TFEQ provided further evidence of the scale's validity. Notably, the ESEBS-BR's two latent scales showed negative correlations with indicators of binge eating and domains of the TFEQ, disinhibition, emotional eating, and cognitive restraint.

## Supplementary Information

Below is the link to the electronic supplementary material.

Supplementary material 1.

## Data Availability

All procedures performed in studies involving human participants were in accordance with the Declaration of Helsinki and approved by the Research Ethics Committee of the Pontifical Catholic University (CAAE: 64659222.0.0000.5336). Informed consent was obtained from all individual participants included in the study.
