# Supplementary material for: Transcultural adaptation and validation of the Eating Self-Efficacy Brief Scale (ESEBS): the Brazilian version
Source: Eat Weight Disord. 2024 Nov 4;29(1):70. doi: 10.1007/s40519-024-01703-2 (PMC11534980; doi:10.1007/s40519-024-01703-2)

**Supplementary material**

**Title: *Transcultural Adaptation and Validation of the Eating Self-Efficacy Brief Scale (ESEBS): The Brazilian version***

***Journal:*** [*Eating and Weight Disorders - Studies on Anorexia, Bulimia and Obesity*](https://link.springer.com/journal/40519)

**Authors: Ana Maria Pandolfo Feoli^1^, Tainá Lopes da Silva^1^, Janete de Souza Urbanetto^2^, Monica D’Amico^3^, Silvia Cerolini^3,4^, Caterina Lombardo^3^**

**1 Eating Behavior Group of the Psychology Postgraduate Program, School of Health and Life Sciences, Pontifical Catholic University of Rio Grande do Sul, Porto Alegre 90619-900, Brazil**

**2 School of Health and Life Sciences, Pontifical Catholic University of Rio Grande do Sul, Ipiranga Avenue 6681 – Partenon, Porto Alegre, RS 90619-900, Brazil**

**3 Department of Psychology, “Sapienza” University of Rome,Via dei Marsi 78, 00185 Roma, Italy**

**4 Department of Human Sciences, Guglielmo Marconi University, Via Plinio 44, 00196, Roma, Italy**

****Correspondence should be directed to:***

**Monica D’Amico**

**Sapienza University of Rome , Department of Psychology**

**Via dei Marsi, 78 – 2nd floor – 00185, Rome, Italy**

**monica.damico@uniroma1.it**

**Caterina Lombardo**

**Sapienza University of Rome , Department of Psychology**

**Via dei Marsi, 78 – 2nd floor – 00185, Rome, Italy**

**caterina.lombardo@uniroma1.it**

**Escala Breve de Autoeficácia Alimentar (ESEBS-BR)**

**(Feoli AM, da Silva TL, Urbanetto JS, D’Amico M, Cerolini S, Lombardo C, 2024)**


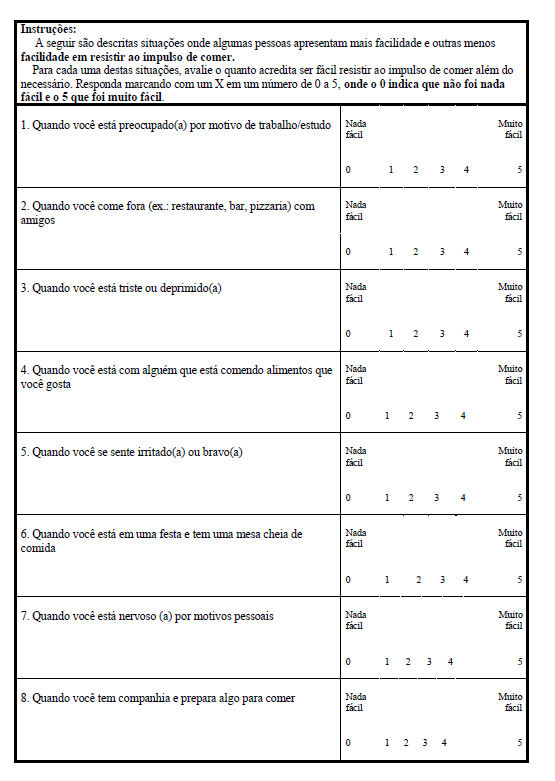

Supplement: Supplementary file 1 — Supplementary material 1. [file 40519_2024_1703_MOESM1_ESM.docx]
